# The Hygienic and Mechanical Treatment of Heart Disease

**Published:** 1902-01

**Authors:** 


### The Hygienic and Mechanical Treatment of Heart Disease.

Dr. Boardman Reed, in *American Medicine*, has an article on the "Hygienic and Mechanical Treatment of Heart Disease." He refers particularly to those forms of heart disease involving auto-toxic degenerative changes in the heart muscle, and to those in which hypertrophy or dilatation has developed without other discoverable cause than faulty metabolism. The heart may by injured by the toxic products of imperfect digestion, suboxidation and other faults of metabolism in at least two ways: (1) The circulating poison may impair the muscle by its direct action; (2) the heart's work may be greatly increased by the contraction of the arterioles, resulting from the action of the alloxuric bodies and other products of imperfect metabolism. Uric acid, certain of the Xanthin bases when present in the blood in excess give rise to contraction of the arterioles. Certain constituents of tea and coffee are closely allied chemically to highly toxic aloxuric bases and are no doubt responsible for many results charged to uric acid. It follows that to prevent threatened cardiac hypertrophy or dilatation and certain renal changes the patient must have a suitable diet, not too nitrogenous, and moderate exercise in the open air. The Norbeim both does good, as do also the artificial carbonated baths to be had in this country. Hot salt water baths employed cautiously, associated with massage and followed by a suitable period of rest yield good results by dilating the superficial vessels. In every form of treatment the patient's pulse should be watched. Especially is this important during the employment of gymnastic movements, since their object is to steady the pulse and diminish its frequency.

